# Supplementary material for: Vegan Nutrition for Mothers and Children: Practical Tools for Healthcare Providers
Source: Nutrients. 2018 Dec 20;11(1):5. doi: 10.3390/nu11010005 (PMC6356233; doi:10.3390/nu11010005)
Supplement: Supplementary file 1 [file nutrients-11-00005-s001.pdf]

nutrients

Supplemental Material

1. The VegPlate

The VegPlate is a plate-shaped food guide which provides an easy method for planning adequate vegan diets both in pregnant and lactating women and in infants and children (Figure S1a) [15,32].

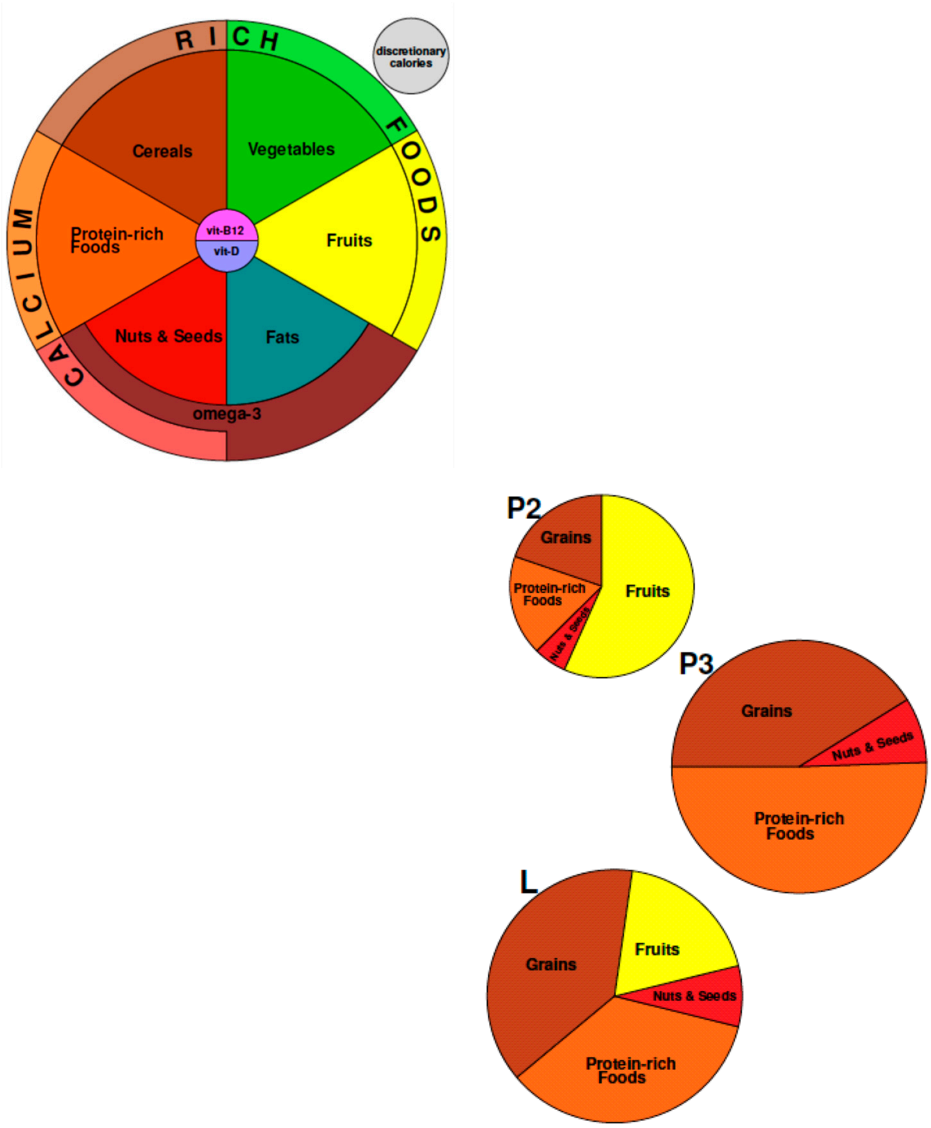

Figure S1. (a) The VegPlate **(b)** graphic representation of the additional servings during the second and third trimesters of pregnancy and during lactation [15, 32].

The VegPlate consists of six main food groups (grains, protein-rich foods, nuts and seeds, vegetables, fruits, fats) plus the two cross-sectional groups of calcium-rich foods and omega-3-rich foods. Vitamin B12 and vitamin D are highlighted in the center of the plate in order to stress their importance in a well-planned plant-based diet.

The adequacy of the diet can be reached during childhood and in the first trimester of pregnancy simply by including daily the suggested number of servings for each food group [15,42]. Additional servings during the second (P2) and third (P3) trimesters of pregnancy and during lactation (L) are required to meet the increased needs of energy and protein (Figure S1b).

The six slices of the main plate are not proportional to the amount, nor to the percentage of daily calories to consume from each food group, as the latter changes according to the different calorie requirements. On the contrary, the slices of the three small plates in Figure S2b are proportional to the relative amount of food to add for each group during these stages of life. The graphic of the main VegPlate (Figure S1a) simply illustrates the variety of plant-based foods to consume daily in a well-planned plant-based diet, and highlights the importance of including good sources of calcium and omega-3 fatty acids and of supplementing vitamin B12 and vitamin D.

During complementary feeding, from 6 to 12 months of age, breast or formula milk still provide most of the energy, calcium, and omega-3 fatty acids requirements [42], and it constitutes the seventh group of the VegPlate (Figure S2). Vitamin B12 and vitamin D are still highlighted at the center of the plate.

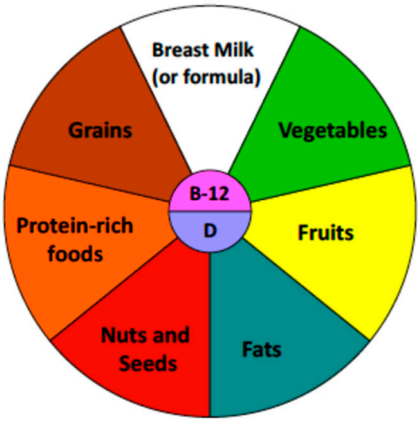

Supplemental Figure 2. The VegPlate for infants (6 to 12 months).

2. Sample Menus

With the VegPlate method, all healthcare providers can easily build adequate vegan diets within a few minutes.

In Table S1 we provide three sample menus.

Supplemental Table 1. Sample menus

	Sample menu of 2500 kcal for a vegan woman in the third trimester of pregnancy	Sample menu of for a 9-month-old vegan infant	Sample menu of 1500 kcal for a 7-year-old vegan child
**Daily numbers of servings from each food-group according to the VegPlate**	Grains (G): 11.5 servings; protein-rich-foods (P): 5 servings; vegetables (V): 6 servings; fruits (F): 2.5 servings; nuts and seeds (NS): 3 servings; fats (f): 3	A fix number of servings from each group is not recommended, but solid meals should be energy-dense and fiber-deprived and contain all the food	Grains (G): 6.5 servings; protein-rich-foods (P): 2 servings; vegetables (V): 4 servings; fruits (F): 2 servings; nuts and seeds (NS): 1.5 servings; fats (f): 5 servings; omega-3-rich foods:

	servings; omega-3-rich foods 2 servings[1].	groups throughout the day. All the	2 servings; calcium-rich-foods 5 servings.
**Breakfast**	Soy yoghurt fortified with calcium (125 g; 1P) with 30 g of unsweetened corn-flakes (1G) and 30 g of oat flakes (1G), 15 g of almonds (0.5 NS), and 15 g of raisins (0.5F).	Breast or formula milk, one rice cake.	Oat milk fortified with calcium (200 ml; 1G; 2 servings of calcium-rich foods), 30 g of whole-wheat bread (1G), and 15 g of tahini (0.5NS; 0.5 serving of calcium-rich foods).
**Mid-morning snack**	Sliced apple (150 g; 1F) with 15 g of sesame butter (0.5NS).	Pureed banana (80 g) with almond butter (10 g), breast or formula milk.	Raspberries (150 g; 1F).
**Lunch**	Quinoa (90 g; 3G) with 160 g of cooked kidney beans (2P), coriander, and 100 g of red bell peppers (1V); 100 g of baby spinach (1V) salad with 30 g of walnuts (1NS; 1 serving of omega-3-rich foods) and dried figs (30 g; 1F) with balsamic dressing and 5 g of olive oil (1f) and 60 g of whole-wheat bread (2G).	Refined rice (25 g, raw weight) overcooked in unsalted vegetable stock with 1 tablespoon of strained pumpkin, hulled red lentils (15 g, raw weight), tahini (10 g), 5 g of olive oil and 5 g of flaxseed oil.	Whole-spelt spaghetti (60 g; 2G) with almonds 15 g (0.5 NS; 0.5 serving of calcium-rich foods) and basil pesto with steamed broccoli 100 g (1V; 1 serving of calcium-rich foods), chickpea 80 g, cooked (1P) and lemon hummus with carrot sticks 100 g (1V), 5 g of olive oil (1f) and 5 g of flaxseed oil (1f; 1 serving of omega-3-rich foods).
**Mid-afternoon snack**	Popcorn (30 g; 1G) with nutritional yeast, 100 ml of celery, kale, and carrot juice (1V) with 75 ml of orange juice (0.5F).	Soy yoghurt fortified with calcium and no added sugars, 80 g with 1 tablespoon of puréed apple, breast or formula milk.	Rice cakes (15 g; 0.5G) with peanut butter 15 g (0.5NS), pear 150 g (1F).
**Dinner**	Pumpkin (200 g; 2V) and cooked chickpeas (80 g; 1P) soup with buckwheat (60 g; 2G), sunflower seeds (15 g; 0.5 NS) and 5 g of flaxseed oil (1f; 1 serving of omega-3-rich foods), spicy roasted tempeh (80 g; 1P) with broccoli (100 g; 1V) and 5 g of olive oil (1f), whole-wheat bread (45 g; 1.5G).	Iron-fortified infant pasta (25 g, raw weight) cooked in unsalted vegetable stock with 1 tablespoon of tomato sauce, split peas (15 g, raw weight), cashew butter (10 g), 5 g of olive oil and 5 g of flaxseed oil.	Sorghum (60 g; 2G) with butter beans (80 g), cooked (1P) and zucchini 100 g (1V), steamed spinach 100 g (1V), 10 g of olive oil (2f) and 5 g of flaxseed oil (1f; 1 serving of omega-3-rich foods).
**After-dinner snack**	Cashews 15 g (0.5NS).	Breast or formula milk.	-

[1]Above 2400 kcal there is no need for counting the servings of calcium-rich foods, as calcium is already provided by the variety of plant-based foods consumed.
